# A Review of Modern Thermal Imaging Sensor Technology and Applications for Autonomous Aerial Navigation

**DOI:** 10.3390/jimaging7100217

**Published:** 2021-10-19

**Authors:** Tran Xuan Bach Nguyen, Kent Rosser, Javaan Chahl

**Affiliations:** 1 School of Engineering, University of South Australia, Mawson Lakes 5095, Australia; Javaan.Chahl@unisa.edu.au; 2 Aerospace Division, Defence Science and Technology Group, Edinburgh 5111, Australia; kent.rosser@dst.defence.gov.au; 3 Joint and Operations Analysis Division, Defence Science and Technology Group, Melbourne 3000, Australia

**Keywords:** review, UAVs, optical flow, simultaneous localization and mapping, SLAM, thermal imaging, LWIR, navigation, neural network

## Abstract

Limited navigation capabilities of many current robots and UAVs restricts their applications in GPS denied areas. Large aircraft with complex navigation systems rely on a variety of sensors including radio frequency aids and high performance inertial systems rendering them somewhat resistant to GPS denial. The rapid development of computer vision has seen cameras incorporated into small drones. Vision-based systems, consisting of one or more cameras, could arguably satisfy both size and weight constraints faced by UAVs. A new generation of thermal sensors is available that are lighter, smaller and widely available. Thermal sensors are a solution to enable navigation in difficult environments, including in low-light, dust or smoke. The purpose of this paper is to present a comprehensive literature review of thermal sensors integrated into navigation systems. Furthermore, the physics and characteristics of thermal sensors will also be presented to provide insight into challenges when integrating thermal sensors in place of conventional visual spectrum sensors.

## 1. Introduction

Research on unmanned aerial vehicles (UAVs) has grown rapidly in the past decade. First, initially developed for military purposes [1], UAVs have been widely used in many applications including industrial inspection [2,3], remote sensing for mapping and surveying [4,5], rescue missions [6,7,8,9,10,11], border control [12] and for other emerging civil applications.

Reliable navigation for autonomous or semi-autonomous operation is essential for these applications. Currently, UAVs rely heavily on an array of sensors for its navigation. Various navigation techniques can be divided into three groups: inertial navigation, satellite navigation and vision-based navigation [13]. The global positioning system (GPS), inertial measurement units (IMU) and barometers are primarily used for determining position, attitude and velocity of the aircraft. However, GPS is known for errors and drop-outs [14] due to signal loss and interference in forests, under tall buildings, in narrow canyons or in remote areas at particular times. IMUs provide a limited period of accurate positioning after external aiding is lost, as they drift without bound from integrating cumulative errors over time [15].

Vision-based navigation systems are a promising research direction in the field of autonomous navigation. Vision sensors can provide real-time information about a dynamic surrounding environment that is resistant to conventional jamming. Vision sensors detect reflected photons or radiated photons in specific bands across the electromagnetic spectrum. Optical sensors perform detection in the visible spectrum that humans can see, while thermal sensors detect infrared wavelength that is invisible to humans.

The predominance of research to date considers optical sensors that require some form of illumination of the scene. There is a substantial gap in the ability to navigate at night given that it has the potential to increase the operational period of vision systems.

## 2. Navigation Problems with Thermal Sensors

Although thermal cameras have been used in visually degraded conditions before, they were mainly used for purposes other than navigation, including: inspection [16,17,18,19,20], crop monitoring and water management in agriculture [21,22,23,24,25]. The main complication that prevents their usage for navigation in natural scenes includes limited features such as edges or textures compared to their visible band counterparts [13]. Furthermore, early versions of thermal sensors included built-in internal corrections that dynamically changed the contrast in the images before output, violating many vision algorithm requirements.

Additionally, early sensors were large, preventing their use on small UAVs. There were also limitations of availability of small and powerful on-board processing hardware. Due to these constraints, many navigation algorithms were initially designed for unmanned ground vehicles (UGV) rather than UAVs. Later, thanks to the introduction of smaller thermal sensors and more capable processing hardware, thermal UAV navigation techniques began to attract interest.

The number of research articles published on this topic has increased in recent years due to the availability of thermal sensors and robotics technology combined with navigation challenges in new applications. However, no single review has yet summarised the relevant articles with a focus on the integration of thermal sensors into navigation systems of UAVs.

### 2.1. Aims and Search Methodology

Considering the observations above, we provide a review with a focus on the integration of thermal sensors for navigation applications within the last decade, from 2010 to the present period. Our paper addresses the hierarchy of issues for a thermal sensor in a navigation system, including the fundamental physics of operation, sensor configurations and computational aspects.

Our method for identifying all relevant papers to include in this study is by using keywords from google scholar and the university library database: “navigation”, “thermal imaging”, “long wave infrared”, “GPS denied”, “deep learning” and “vision-based techniques.” The range of papers according to their year of publication is from 2010 to 2021. The selected articles were then divided into different categories based on the type of algorithms used. Furthermore, sensor specifications and configuration aspects will be discussed in order to analyse which navigation applications can be achieved and the performance of each system.

### 2.2. Structure of the Paper

The paper is organised into thirteen sections. Section 3, Section 4 and Section 5 will focus on the development of commercial thermal sensor technologies, the physics concepts and the sensor configurations for different navigation applications.

Section 3 considers the thermal sensor developments in the last 10 years, from the oldest to the most recent studies. Section 4 introduces the fundamental concepts behind the electromagnetic and infrared spectra. After that, Section 5 will highlight some important features of thermal sensor configurations, including sensor calibration and the relevant aspects of built-in correction techniques.

After discussing hardware characteristics of thermal sensors, Section 6 presents the basic concepts of different algorithm types for vision-based systems. Section 7 presents works in Simultaneous Localisation and Mapping (SLAM). Section 8 presents works in optical flow, and Section 9 reviews works in neural networks.

Section 10 discusses the various roles used for thermal sensing in navigation, while Section 11 describes the difference in system requirements for the thermal image in different navigation approaches.

Section 12 and Section 13 present the discussion and our observations about future research directions of the field.

## 3. Thermal Sensor System Considerations for Navigation Applications

Although the history of thermal sensor technologies is well described in [26], it is worth considering the sensors in the context of navigation sensing in small to medium autonomous vehicles, particularly those that are airborne. This section will discuss the specifications of different thermal sensors that are suitable for navigation application, including cooled and uncooled sensor technologies, dimensions, weights, power consumptions, resolutions and effective frame rates.

Table 1 shows the specifications of all of the thermal sensors appearing in work we have reviewed from the last 10 years for navigation applications.

### 3.1. Cooled and Uncooled Sensor

A major practical classification of thermal technologies is cooled vs. uncooled sensors. A cooled thermal sensor has an integral cooling system to lower the sensor temperature to cryogenic temperatures (120 K or −153 ${}^{{}^{\circ}}$C) in order to achieve a higher signal to noise ratio (SNR), thereby allowing higher thermal sensitivity, higher spatial resolution and higher frame rates. However, cryocoolers typically contain mechanical parts, produce far more heat on the other side of circuit, which contributes to larger size and weight, and reults in high power consumption of the imaging device. These characteristics might be tolerable in large vehicles, but such devices are likely to exceed space, weight and power (SWAP) capacities of smaller multirotor and hand launched drones.

Uncooled sensors, on the other hand, are smaller in both size and weight at the cost of inferior all-around performance. However, the study in [31], which compared a high-end cooled FLIR Phoenix to the more affordable uncooled system Variocam, showed that the uncooled thermal system could compensate for its lower resolution sensitivity via further image processing. Furthermore, the study also showed that the uncooled thermal sensor was still suitable for use in UAVs for navigation applications by analysing SNR data. It has been observed that all of the thermal sensors used for commercial mass-market purposes in the last decade have been uncooled.

### 3.2. Sensor Specification Constraints for Unmanned Platforms

Earlier thermal sensors were heavy and bulky. The earliest study consisted of a 4.54 kg thermal sensor and was conducted on UGVs where size and weight were tolerable. For small and medium commercially available quadrotor drones, the recommended payload limit is usually less than 800 g. For example, the DJI Phantom 3 and Phantom 4 can safely carry a payload of 700 g and 800 g, respectively [32]. When considering the entire payload, it will include other components such as onboard computers, batteries and other sensors and the size and weight of the thermal sensor alone that require substantial reduction from early airborne implementations.

Other factors include effective frame rate, resolution and power consumption, which are related to each other. Higher frame rate and resolution translate to higher power consumption for the system overall. Higher frame rate and resolution require more computational effort from the onboard computer combined with higher power demand from the sensor itself, resulting in a bigger battery and a bulkier system. For highly dynamic systems, however, there is no substitute for frame rate.

In addition to flying platforms, when selecting a suitable sensor, the type of navigation algorithm is also a crucial factor. Map building algorithms such as SLAM require higher frame rate, higher resolution and are more computationally expensive. While the map-less techniques do not require very high resolution and frame rate and are cheap to execute, they tend towards navigation in relative terms rather than absolute. The details of the two approaches will be presented in Section 6.

Recent sensors from FLIR such as the FLIR Tau2, Boson and Lepton have weights and sizes significantly lower while still maintaining resolution and frame rate. Furthermore, the prices of the recent thermal sensors such as the FLIR Boson and the FLIR Lepton have become more attainable for research compared to the previous generation of sensors. Consequently, more researchers have begun to integrate thermal sensors into UAV navigation systems.

### 3.3. Platform Considerations

Different UAVs fit different missions, which requires different system configurations, size and algorithms [33,34]. For example, for a search and rescue or survey mission in a confined area such as underground in mines or in urban areas, a multirotor is a good choice due to its size and maneuverability. The multirotor tends to be smaller than fixed-wing aircraft and is not used for long distance transits. They have a major advantage in their ability to stop and hover, which is a very specific navigation state resulting in behaviour focused on maintaining a stationary image and constant height.

On the other hand, for missions such as border control, search or survey over large open areas, a fixed wing aircraft can be a better choice than a quadrotor due to its long operating time and superior range. In defense applications, fixed wing aircraft are more often used due to their long range and endurance, high operating ceiling and the ability to carry large payloads of sensors and weapons. Fixed wing UAVs require more complicated take off and landing procedures, are more challenging to operate, are less compact and range in size from that of a human hand to a passenger airliner. With their need to stay in forward flight and requirement to fly longer distances, their navigation behaviour is focused on trajectories, wind and forward movement.

## 4. Physics of Thermal Sensors

Thermal sensors operate differently than optical sensors. Unlike optical sensors, thermal sensors rely on emissions of longer wave infrared radiation rather than reflection of shorter visual wavelengths. While thermal infrared wavelengths can reflect off surfaces and images captured using thermal sensors can be somewhat affected by the surrounding environment, this is a minor effect in general. Given the emission driven mode of the sensor, it is essential to examine the physics of thermal sensors and emissivity values of different materials.

This section briefly introduces the fundamental concepts behind infrared sensors, including black body radiation theory, the electromagnetic spectrum and the emissivity value of different materials. All related concepts are well described in [35,36].

### 4.1. Black Body Radiation

A black body radiator is an object at near thermodynamic equilibrium that absorbs and emits all radiation frequencies [37]. At near thermodynamic equilibrium, the emitted radiation or thermal radiation can be expressed by Planck’s law [38]. Planck’s law expresses the spectral radiation emitted by a black body at thermodynamic equilibrium:

(1) $$\beta \left(\nu ,T\right)=\frac{2\hbar \nu^{3}}{c^{2}}\frac{1}{e^{\left(\nu \hbar /kT\right)}-1}$$

where

- β(ν,T) is the spectral radiance of the object at temperature T(K) and frequency ν;
- *ℏ* is the Planck constant;
- *c* is the speed of light in vacuum;
- *k* is Boltzmann’s constant;
- ν is the frequency of the electromagnetic radiation;
- *T* is the absolute temperature of the object.

When the temperature of a black body is at several hundred degrees Kelvin, most of the emitted radiation is infrared. When the temperature is higher, it is emitted at shorter wavelengths which are in the visible region.

### 4.2. Electromagnetic Spectrum

**Figure 1:**
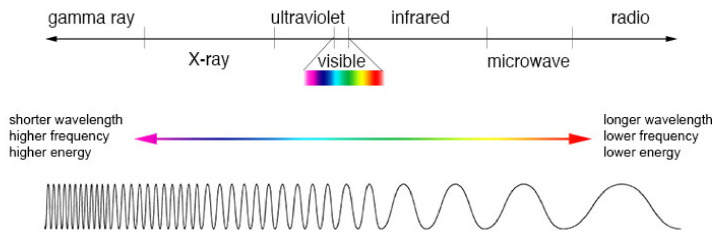
Comparison of wavelength, frequency and energy in the electromagnetic spectrum [40] (credit: NASA).

Figure 1 shows different wavelengths in the electromagnetic spectrum. Radio and microwaves lie at the longer end of the spectrum of electromagnetic energy, while gamma ray and X-rays have very short wavelengths. Humans can only see a limited range of the spectrum from 380 nm to 700 nm [39].

Infrared radiation was discovered in 1800 by William Herschek [41]. Infrared is the part of the electromagnetic spectrum from 8 to 15 μm [42]. Most of the energy in this spectrum is radiated as heat and can be observed both during the day and night. Since the infrared spectrum has longer wavelength than visible light, it is less attenuated by denser mediums such as vapour, dust or smoke [43]. This paper will focus on applications of the infrared spectrum.

### 4.3. Emissivity

Emissivity of an object is a measurement of its ability to emit thermal energy [44]. Emissivity of 0% is a perfect thermal mirror that reflects all infrared energy, and 100% is a black body that absorbs and radiates all energy [38].

Table 2 shows the emissivities of some objects, both metal and non-metal, including polished or oxidised/roughened metal. It is clear that polished non-oxidised materials have lower emissivity values compared to oxidised materials. Non-metallic materials such as glass and water have a high emissivity value; thus, infrared wavelengths do not penetrate glass or water.

It is also apparent from Table 2 and the principle of black body radiation that thermal imaging is substantially different from optical imaging. The low emissivities of some manufactured surfaces but relatively high emissivities of natural surfaces show that thermal imaging devices will tend to observe scenes through radiation rather than reflection. Objects radiate energy absorbed from the sun earlier and reflect thermal radiation for other objects and the ground at quite modest levels unless they are finished metal surfaces. Thermal scenes are usually equivalent to scenes composed of light sources if they were in the optical domain, which has implications for how and why they are used for navigation.

**Figure 2:**
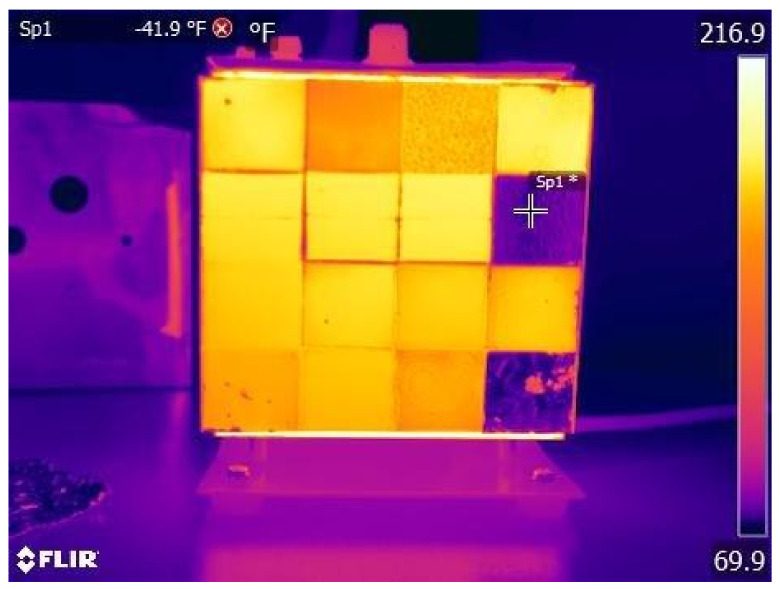
Sample of infrared radiation of different materials [46].

Figure 2 shows an example from FLIR [46] showing that different materials, metal and non-metal, emit infrared radiation due to different emissivity values at the same temperature.

In addition to the material of emitting objects, other factors that should be considered include the temperature, humidity, reflective surfaces and convection airflow of the intended operational environments [47]. This has substantial implications for navigation in natural environments. Cloud, rain and dust are likely to affect optical and thermal cameras.

In vision-based navigation algorithms, textures, corners and edges in the image are vital requirements. Hence, a thermal system will not provide much information in a uniform temperature scene, such as water, snow or sand. Conditions with little temperature variation between day and night and with minimal solar radiation during that day might have very little thermal contrast in scenes that are imaged even if the scenes are not a uniform material.

The study by [48] utilised this concept by applying thermal markers made of thin 5 mm acrylic sheets over objects in smoke filled environments. Due to high emissivity values of acrylic at 0.88 [49], the thermal markers are distinct in the thermal frame, which later could be used for map-building SLAM techniques.

## 5. Thermal Sensor Configurations

Sensor configurations have a direct effect on how data are collected and processed. This section details the crucial concepts of thermal sensor calibration and their impact on vision-based navigation. Problematically for autonomous system applications, modern thermal sensors have external and internal correction techniques that are designed for human visualisation and inspection purposes rather than computer vision, and this difference and its implications are discussed in this section.

### 5.1. Sensor Calibration

Geometric calibration is an essential task when using a combination of cameras in navigation applications. Chessboard is a standard method for optical camera calibration [50,51,52], but when translated to thermal cameras, heated lamps or using material with different emissivity values, it not only makes the task more complex but also inaccurate and expensive [53,54].

The authors in [55,56] found that the calibration results significantly vary between tested thermal cameras. Furthermore, cameras have shown very large decentering distortions and deviations in both image coordinate axes.

A trinocular vision system [57] of one optical camera and a thermal camera was calibrated with a plastic board with 25 holes with 25 miniature bulbs arranged at each hole to emit heat and light. This method successfully obtained the required calibration parameters for the thermal sensor for their application. Vida et al. proposed that a geometric mask-based approach could obtain an improvement of 78% compared to the conventional method of a heated chessboard [58]. The work described an A2 board that was built from Medium Density Fibreboard (MDF) due to its good thermal insulation properties, and although it required substantial warming time, the part was laser cut with 40 × 40 mm squares across the board.

### 5.2. Re-Scaling and Correction Techniques

Thermal infrared sensors typically sample radiometric data at 14 bit [59] resolution, while modern electronic display standards and many computer vision libraries require an 8 bit input source. As a result, many previously proposed approaches either rely on Automatic gain control (AGC) or normalisation to convert 14 bit raw data to 8 bit.

When converting from 14 bit to 8 bit format, there is a 6 bit loss of information and a reduction in dynamic range which degrades performance in many circumstances. The study in [60] shows that, while using the same algorithm, using full radiometric thermal information as inputs produced better performance than a re-scaled version. They also showed that using re-scaled data may accumulate errors over time, resulting in thermal data being presented incorrectly.

#### 5.2.1. Automatic Gain Control

**Figure 3:**
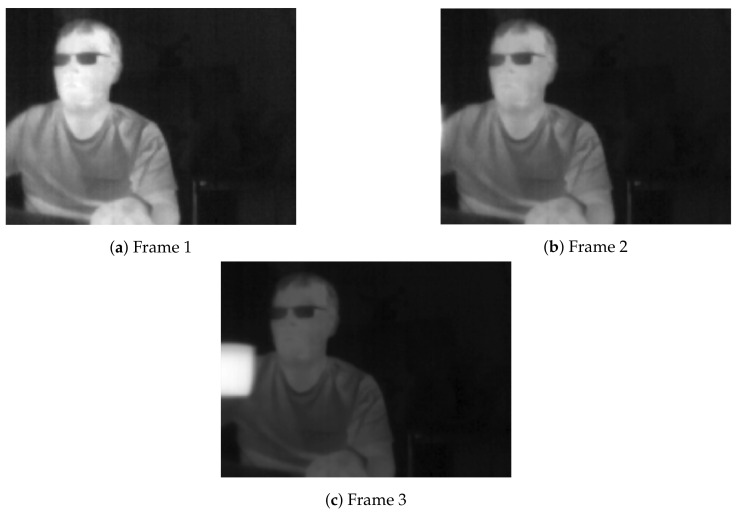
Change of contrast caused by AGC when a hot object moves into a scene.

Many thermal sensors rely on Automatic Gain Control (AGC) to convert raw radiometric data to usable 8 bit input for modern standard displays. The main purpose of AGC is to improve the contrast of an image based on the radiometric range in an observed scene when converting to 8 bit depth. The AGC technique is usually applied by default when there is a change in scene temperature due to hot or cold objects exiting or entering the field of view. A practical example shows that it comes at a cost: A substantial change in overall brightness in two consecutive frames is observed when a hot cup moves into a scene, as shown in Figure 3. The contrast change is likely to cause problems for many image matching techniques that are fundamental to visually aided navigation due the drastic changes in image appearance and the likelihood that some information might be discarded between frames. Although 8 bit processing seems like an arbitrary concern, the vast amount of software is written with 8 bit processing in mind, and hardware acceleration libraries make it a non-trivial consideration.

To solve this problem, some work-around methods have been proposed. The authors in [61] reduced the response time of the AGC so that the brightness does not change rapidly in order to perform matching of the features. However, this approach only reduces the problem and does not completely solve it. It also creates errors in image processing techniques that use spatiotemporal gradient information. Another approach is to set the range for the AGC while operating via some external means or manually [62]. Nevertheless, this requires prior knowledge of the environment or a new set of scene analysis logic, making it less adaptable to unknown environments.

Rosser et al. re-scaled one pair of 14 bit images at a time to work with feature detection for optical flow [63]. The technique works particularly well in OpenCV [64] by using Shi–Tomasi corner detection [65] and the Lucas–Kanade optical flow technique [66]. The technique is based on the maximum and minimum pixel intensities in a pair of images, which will be explained in Section 8.1.

### 5.3. Flat Field and Non-Uniformity Corrections

A correction technique to fix accumulated pattern noise over time in thermal sensor systems is Flat Field Correction (FFC) for sensors with a shutter or Non-Uniformity Correction (NUC) for sensors without a shutter. During operation, the FFC/NUC freezes the sensor for a small amount of time (0.3–2 s), depending on the camera model, in order to correct for errors. This operation is essential for stationary applications where the sensor captures the same scene for a long time. However, it comes with a downside of occasional data interruption which is potentially undesirable for navigation applications. Vidas et al. designed a thermal odometry system that performed NUC only when needed depending on the scene and pose [67].

On the other hand, in some recent sensors such as the FLIR lepton 3.5 [59], a built-in internal calibration algorithm that is capable of automatically adjusting for drift effects can compensate for FFC/NUC for moving applications. As described in studies in [63], the FFC was not needed since the sensor was mounted on constantly moving aircraft.

## 6. Vision-Based Navigation Systems

Vision-based systems rely on one or more visual sensor to acquire information about the environment. Compared to other sensing systems such as GPS, LIDAR, IMUs or conventional sensors, visual sensors obtain much more information such as colours or texture of the scene. The available visual navigation techniques can be divided into three categories: Map based, Map building and Mapless systems.

### 6.1. Map Based Systems

Map based systems rely on knowing the spatial layout of the operating environment in advance. Hence, the utility of this type of system is limited in many practical situations. At the time of writing, there is no proposed work with thermal cameras.

### 6.2. Map-Building Systems

Map-building systems build a map while operating, and they are becoming more popular with the rapid advancement of SLAM algorithms [68]. Early SLAM systems relied on a system of ultrasonic sensors, LIDAR or radar [69]. However, this type of payload limits their use in small UAVs. Therefore, more researchers have shown interest in single and multiple camera systems for visual SLAM. Related works will be presented in Section 7.

### 6.3. Mapless Systems

A mapless navigation system can be defined as a system that operates without a map of the environment. The system operates based on extracting features from the observed images. The two most common techniques in mapless systems are optical flow and feature extracting techniques. The related works will be presented in Section 8.

## 7. Simultaneous Localisation and Mapping

Simultaneous Localisation and Mapping (SLAM) is a mapping technique for mobile robots or UAVs to generate maps from operating environments. The generated map is used to find the relative location of the robot in the environment to achieve appropriate path planning (localisation). The first SLAM algorithm was introduced in [70], where they implemented the Extended Kalman Filter technique EKF-SLAM.

In early works, many different types of sensor such as LIDAR, ultrasonic, inertial sensors or GPS were integrated into the SLAM system. Montemerlo et al. [71] proposed a technique named FastSLAM, a hybrid approach utilising both the Particle Filter and Extended Kalman filter techniques. The same team later introduced a more efficient version: FastSLAM2.0 [72]. Dellaert et al. [73] proposed a smoothing technique called Square Root Smoothing and Mapping (SAM) that used the square root smoothing technique to solve the SLAM problem in order to improve the efficiency of the mapping process. Kim et al. [74] proposed a technique based on unscented transformation called Unscented FastSLAM (UFastSLAM), which is more robust and accurate compared to FastSLAM2.0.

Recently, SLAM system using cameras are actively explored with the hope of achieving reduced weight and system complexity. Since SLAM takes only visual images as input, it is referred to as visual SLAM (vSLAM) [75]. Various low computation techniques have been proposed in the literature that are suitable for UAVs with limited resources onboard. A typical SLAM application for small UAVs is visual odometry.

### 7.1. Combined Spectrum Techniques

In early works, some authors tried to utilise both the LWIR and visible spectra to enhance or mitigate features that were hidden due to external factors such as fog or smoke.

Maddern and Vidas [76] proposed a technique to combined 8 bit thermal with RGB images for UAV navigation. The study showed that there are extreme changes in the visible spectrum during the day and night, while the thermal spectrum remains consistent but with lower contrast over time. The combined spectrum produced better results with algorithms that used visual or thermal frames alone. Poujol et al. [77] showed that combining visual and thermal spectrum can greatly improve the performance of classic visual odometry approaches. The study used two image fusion techniques: monochrome threshold based image fusion [78] and monocular visual odometry [79]. The data were collected from an electric vehicle moving around a city. The experimental results show that the fused images could provide extra data to achieve more robust solutions.

Brunner et al. [80] presented a preliminary evaluation study of combining optical and thermal cameras for localisation in an environment filled with smoke or dust for autonomous ground vehicle (AGV). The study showed that relative motions could not be estimated from visual images in that environment, while motions can be estimated from thermal images but with less accuracy. The authors in [81] proposed a technique to combine both LWIR and the normal spectra in order to enhance a VSLAM algorithm by rejecting low quality images that may have introduced localisation errors. The technique was tested in several adverse conditions such as smoke, fire, at dusk and in low light conditions that have unfavourable effects on both the thermal and visual spectra.

A flexible SLAM network described in [82] utilised both thermal and visual information to build a colour map of the environment under low illumination environments. Multispectral stereo odometry from optical and thermal sensors was introduced in [83] for a ground vehicle. Khattak et al. [84] relied on a combination of radiometric sensors, the FLIR Tau2 and a visual camera to create the navigation capacity for a small quadrotor in an indoor dark and dust filled environment. An Intel NUC-i7 computer (NUC7i7BNH) was installed in the UAVs to perform all the calculation tasks onboard. Thermal frames enabled robust feature selection combined with an Extended Kalman Filter for odometry estimation by the drone. The study showed that the thermal sensor helped the fusion system to work reliably in low visibility environments.

### 7.2. Thermal Spectrum Techniques

This section presents work and algorithms that use thermal sensors as the only source for collecting data, which can be divided into two categories: techniques that use 8 bit re-scaled data or work that makes use of higher bit depth radiometric data.

#### 7.2.1. Re-Scaled Data

Mouats et al. [61] also developed a thermal stereo-odometry for UAV applications based on localisation solutions from a pair of thermal images. The authors used a pair of re-scaled 8 bit images with applied AGC and the FFC turned off. To compensate for the sudden change in contrast from the AGC, they employed a technique to reduce response time for the AGC so that the matching feature algorithms could still function. However, this made the algorithm less adaptive to the environment.

Another study by Khattak et al. [48] used a LWIR sensor alone to detect low thermal conductivity fiducial markers in order to localise in a dark indoor scene. The team attached a thermal fiducial marker to fixed objects around the environment in an incremental manner. The new marker was observed at the same time as previously predefined ones. The poses and the coordinates of the platform estimated from this method showed it to be on par with the ground truth Inertial Measurement Unit (IMU).

The ROVIO [60] algorithm was shown to work well with re-scaled 8 bit images in indoor environments. The algorithm was modified to work with full scale radiometric data, named ROTIO. The ground truth was provided by a motion capture system. The result shows the advantages of using full radiometric data. The FFC was turned off to prevent tracking loss due to data interruption.

#### 7.2.2. Full Radiometric Data

Shin and Kim were the first to propose a thermal-infrared SLAM system using measurements for 6-DOF motion estimation from LIDAR on full radiometric 14 bit raw data [85]. The experimental results show that the 14 bit system overcame the limitation of the re-scaling process and was more resilient to data loss. Moreover, relying on full radiometric data, Khattak et al. [86] proposed a thermal/inertial system that utilised the full range of radiometric data for odometry estimation. The study showed that using full radiometric images was more resilient against loss of data due to sudden changes caused by the AGC re-scaling process.

Although the previous works show promising outcomes, the SLAM algorithms are computationally demanding and many require high resolution thermal images. Many aforementioned works use high resolution thermal cameras such as the FLIR Tau2, which costs thousands of dollars. Furthermore, a compact yet powerful onboard computer system is also expensive in terms of money as well as space, weight and power. All of these are difficult challenges for integration into small UAVs.

## 8. Optical Flow

Optical flow is a map-less measurement technique defined as the pattern of apparent movement of brightness across an image [87]. Optical Flow can be used in navigation solutions that have been inspired from insects such as the honeybee [88]. The honeybee navigation system relies on optical flow for graze landing [89,90] and detecting obstacles avoidance [91]. Unlike SLAM, optical flow algorithms require much less computational resources and do not require very high resolution input images. Additionally, optical flow algorithms, such as the sparse Lucas–Kanade technique in OpenCV, are known for their efficiency and accuracy for many applications [63,92,93,94,95,96,97]. Hence, optical flow based systems can satisfy both weight and size constraints for integration into small UAV navigation systems.

### 8.1. Thermal Flow

**Figure 4:**
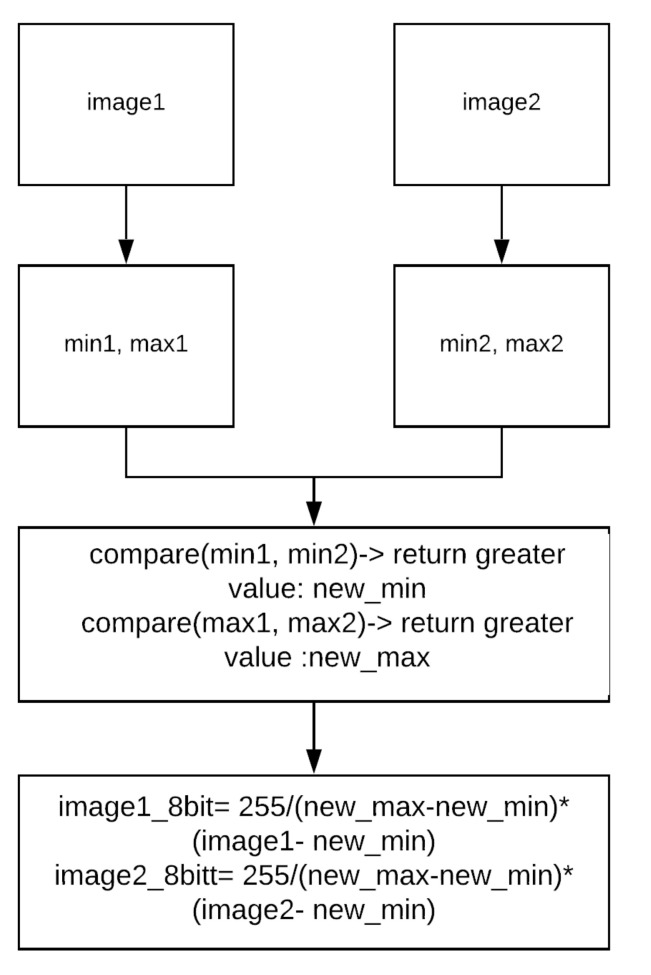
A technique to re-scale from 14 bit to 8 bit images based on max and min pixel intensities [63].

The term “Thermal FLow” (TF) applies to LWIR-based flow sensing. Rosser et al. proposed a technique to calculate optical flow from re-scaled 8 bit thermal data [63]. Optical flow estimation operates based on several assumptions, including brightness consistency across two images. However, due to the effect of the AGC when re-scaling to 8 bit, there is a violation of this crucial requirement. Hence, the authors came up with a technique to preserve contrast for each pair of images as shown in Figure 4.

In their works, thermal flow relied on the Pyramid Lucas–Kanade [66] algorithm in OpenCV [98]. Good tracking points were found based on the Shi–Tomasi corner detection algorithm [65]. Table 3 shows settings for parameters of the LK and tracking functions in OpenCV.

**Figure 5:**
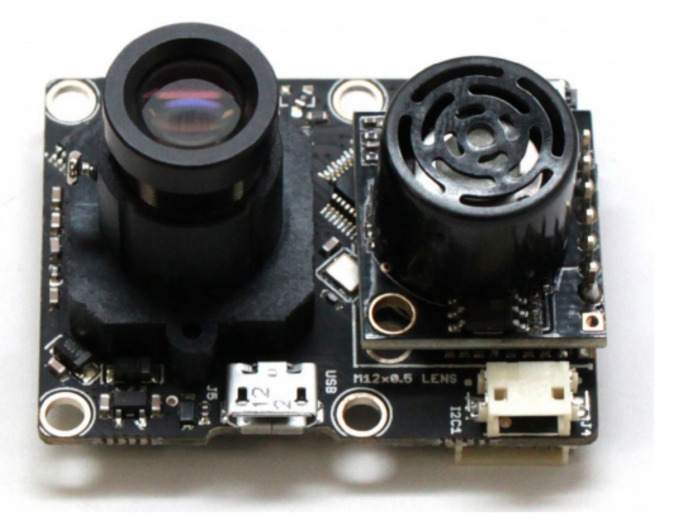
The PX4FLOW optical flow sensor with mounted lens on the left and ultrasonic sensor on the right [93].

They built a low-cost system consisting of a Raspberry Pi with a low-resolution radiometric FLIR sensor Lepton 3.5. The low cost system was designed to mimic the operation of the PX4Flow [93], a low cost and light weight optical flow sensor system, as shown in Figure 5. The sensor was designed to produce reliable two dimensional flow vectors for small hovering platforms such as pocket drones [99,100,101], quadrotor [99,102] and small fixed wing aircraft [92]. Due to the PX4Flow sensor operating in the visual spectrum, its operation is heavily compromised in low light conditions but it was useful as a ground truth to evaluate the thermal flow sensor performance during the day.

**Figure 6:**
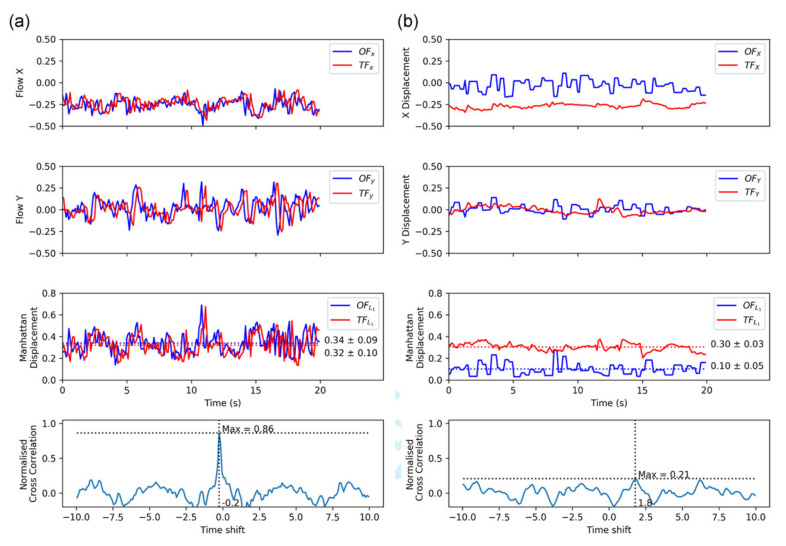
Optical flow (OF) and thermal flow (TF) comparison during (**a**) day and (**b**) night flights. (**a**,**b**) Each include four plots. From the top to bottom sections are the following: OF and TF X-axis angular displacement (radians/second); OF and TF Y-axis angular displacement (radians/second); OF and TF Manhattan distance angular displacement; and Normalized cross-correlation of the OF and TF Manhattan distance displacement signals (dimensionless). Results for the day flight shows high correlation between optical and thermal values, while the night flight shows low correlation as visible spectrum OF is degraded in low light [63].

Rosser et al. [63] mounted the payload on a fixed wing aircraft to reduce lateral drift. The aircraft took two flights, one during the day with sufficient illumination and one later in the night after dark. The results showed that the TF system operated equivalently compared to the optical flow sensor during daylight while also being able to operate at night. Figure 6 shows the results from this study.

The results in Figure 6a show that thermal flow works comparatively well compared to optical flow in the X and Y directions. The third plot shows the Manhattan displacement for both, which show that the signals from OF and TF are closely matched. The fourth plot indicates a strong normalised cross correlation value of 0.86 showing the two signals represent the same phenomenon.

The results in Figure 6b show that TF works to some degrees at night, and OF obviously does not work. The Manhattan displacement and low cross correlations signals at 0.3 indicate that the signals are no longer similar. It can be concluded that TF performs well during the day and still works to some degree during the night.

## 9. Deep Learning

Deep learning and neural networks are the primary choice in many vision tasks since the introduction of the convolutional neural network (CNN) architecture, AlexNet [103]. AlexNet won the large scale visual recognition competition (ILSVRC) by a large margin. As a result, many researchers have since applied CNNs to many computer vision tasks with great success.

### 9.1. Thermal Image Enhancement

Choi et al. proposed a neural network to enhance low resolution thermal images [104]. The network was inspired from a RGB counterpart from [105] but with much less computational demand. The network consisted of three convolutional layers to extract a set of feature maps, followed by the last layer to combine the predictions to reconstruct the high resolution output.

The study used the RGB training dataset from [106] for the entire training process. During the testing phase, a test dataset consisting of both RGB and thermal images from [107,108,109] was used. The model was evaluated in three different scenarios, pedestrian detection, image registration and visual odometry, which showed that the proposed technique was not only capable of reproducing higher resolution images but also with lower noise and fewer unwanted artifacts.

### 9.2. Deep Learning Neural Network Based Odometry

Saputra et al. [110] are the first to propose a DNN-based odometry architecture using thermal images as input. They proposed a DNN-based method for thermal-inertial odometry (DeepTIO) using hallucination networks. They modified an existing state-of-the-art neural network that uses visual images as input into a Visual/Inertial Odometry (VINet) model [111] combined with selective fusion [112]. Since radiometric data contain only one channel, two extra channels were duplicated from the first one, resulting in three channels for the neural network. To provide missing information, a hallucination network was implemented to provide complementary information. The model consisted of the following: a feature encoder, a selective fusion module and a pose regressor.

Hand-held thermal data were collected with FLIR E95 operates at 60 fps with 464 × 348 resolution to collect data in five different buildings, some filled with smoke. Furthermore, a FLIR Boson with 640 × 512 resolution, placed on a mobile robot operated in different testing rooms with various obstacles and lighting conditions. The results show that DeepTIO outperformed VINet in most scenarios. In particular, the performance of VINet decayed when there was insufficient illumination while DeepTIO could still produce reliable and accurate trajectory. However, the DeepTIO network could only work well at 4–5 frames per second; anything lower or higher resulted in a decrease in accuracy. While this is not explained, this indicates some effect caused by camera noise and change in images caused by platform motion.

## 10. Roles of Thermal Sensors in Navigation Systems and Applications

This section discusses the role of thermal sensors in the literature in order to explore how thermal systems have evolved in the last 10 years.

Early use of thermal sensors in robotics saw sensors mounted on UGVs due to their size and weight. The earliest relevant paper was conducted on a UGV [80]. Later, with the introduction of lighter and smaller sensors, UAVs have been the primary choice throughout. Ultimately, there are cheaper and higher resolutions options for UGVs, such as carrying illumination (head lights) for optical sensors.

Initially, researchers explored the possibility of combining both the visual and infrared spectra in normal conditions where the optical sensor would still operate. The role of thermal sensors in this case was to identify and reject bad batches of data [76,81]. Later, the thermal sensor system was integrated to be used in reduced illumination conditions when optical sensor performance was degraded. Thermal sensors played a bigger role in this scenario where thermal data were fused with optical data to construct 3D environments and visual odometry [82,83]. Additionally, thermal data were used to correct and compensate for missing data from the optical sensor due to lack of illumination in the environment [84].

Later, thermal sensor based navigation systems were experimented on in total darkness or visual degraded environments, such as rooms filled with smoke and dust where the optical sensor could not reliably capture the scene. In this case, thermal data were the only input for navigation algorithms. At first, re-scaled 8 bit thermal data were utilised for stereo odometry [61,83], SLAM [48] and optical flow [63]. The disadvantages of 8 bit re-scaled data have been discussed in Section 5.2.1.

In deep learning, neural networks and mapless algorithms [63,104,110], only 8 bit data were used, and no work has been performed to explore the possibility of full radiometric data in this field yet. It is likely that this is partly driven by the availability of appropriate software platforms.

With the introduction of radiometric sensors, some groups have been experimenting with adapting their works to include full resolution thermal data at 14 bit. Results in in [60,85,86] have shown that full radiometric 14 bit thermal data can improve the consistency of the algorithm over time and overcome the need for AGC correction techniques.

## 11. Navigation Approaches with Respect to System Configuration

There are two main navigation approaches found in this survey of the literature: VSLAM and variations on odometry. The former can provide an absolute navigation solution, and the latter provides a dead reckoning solution that is relative to a starting point. The former, when it fails, is likely to result in catastrophic navigation failure with the navigation filter diverging or finding a wrong solution (in the absence of other safeguards), and the latter inexorably drifts over time.

Due to the nature of map-building techniques, higher resolution and frame rate thermal sensors are more desirable due to the need to detect specific features. Hence, it has been observed that map-building techniques relied on better thermal sensors than map-less techniques.

### 11.1. VSLAM

Brunner et al. [80] conducted a study to investigate the use of thermal images to compensate for missing and erroneous information in RGB images due to external adverse lighting effects. The same group in [81] later developed a framework to extract matching features from thermal data. Thermal data could compensate for the missing parts of an RGB image due to smoke or dust. The result demonstrated that the technique greatly improved SLAM performance. In the study, a low frame rate Raytheon Thermal-Eye 2000B thermal system was mounted on the UGV used to capture thermal frames at different times during the day. A lower frame rate of 12.5 fps was sufficient in this case due to the slow movement of the platform, and the framework was not intended for real-time use.

Later, the study in [82] developed a faster and more accurate framework for feature extraction and matching SLAM. The framework was shown to be less computationally demanding while achieving better accuracy. The thermal system specifications are not known due to the authors’ reliance on their own dataset.

The authors in [85] integrated LIDAR into the thermal system to introduce depth information into their LIDAR SLAM system. The team also used a radiometric FLIR A65 sensor with full 14 bit thermal data in their experiment. The SLAM algorithm was modified to work with 14 bit thermal data and demonstrated a better overall performance compared to previous 8 bit SLAM techniques.

### 11.2. Odometry

The team in [83] proposed a combined spectrum of visual odometry for ground vehicles. The study used several thermal and optical sensors in their system configuration to collect data. Log-Gabor wavelets and the optical LK algorithm were used for feature extraction and matching. The study showed that thermal data can enhance the performance of the stereo-odometry system.

Later, the same team adapted their techniques for UAVs with a lighter and smaller thermal sensor, the FLIR Tau2. The study relied on the 8 bit processed thermal frames. To overcome the troublesome AGC correction, the AGC threshold and reaction time was modified to prevent sudden change in image contrast. This modification has proven to work with their collected dataset but did not completely solve the problem.

Using the same sensor, the same group further performed three studies on odometry in order to estimate the state of UAVs. Firstly, the authors [84] utilised 8 bit thermal data with the extended Kalman filter framework for feature extraction and matching. Later, they experimented with thermal markers [48] with very high emissivity, as described in Section 4.3. The thermal markers were applied throughout the test site as distinctive landmarks that are visible in thermal frames that could be used for odometry estimation.

In their most recent study [86], the team proposed a thermal-inertial odometry estimation framework utilising full thermal 14 bit data. The odometry of the platform was estimated from the fusion of inertial measurements and jointly minimized the reprojection errors of 3D landmarks and inertial measurement errors. The 14 bit system was shown to be more resilient to the loss of information caused by the re-scaling process.

### 11.3. Other Applications

Rosser [63] introduced the concept of thermal flow, which is optical flow estimation from thermal imaging. They introduced a bit-depth conversion process that maintained contrast across a pair of images Section 8.1. Due to the nature of optical flow algorithms and odometry applications, a lower resolution and frame rate thermal sensor was still sufficient. This is also the only study that used a fixed-wing aircraft.

In the important domain of deep learning, Saputra et al. [110] utilised the best commercial thermal OEM modules on the market, the FLIR Boson and the FLIR E95, at a frame rate at 60 fps for data collection.

Finally, Table 4 shows a summary works reviewed in this article.

## 12. Discussion

Despite thermal cameras being available for research use for many years, there appears to have been limited efforts to date with thermal imaging for UAV navigation. One of the reasons is the popularity of very low cost GPS receivers despite the challenge presented when it is unavailable.

The cost of thermal sensors is prohibitively high compared to visible light cameras. For example, FLIR Tau2 costs almost $5000 in June 2021 [113]. Hence, it can greatly increase the initial cost of a research project or dramatically increase the cost of a commercial system.

High performance thermal sensors such as the FLIR Tau2 and the FLIR BOSON are under export control by the United State Department of Commerce [114]. For example, all uncooled thermal sensors with frame rates above 9 Hz can only be exported to Strategic Trade Authorized (STA) countries. Some territories are completely barred from importing any thermal systems, while for other countries the sensors are modified to lower frame rates. Lower frame rates are undesirable for aerial applications because many algorithms expect frame rates higher than the dynamics of the system.

Night operations of UAVs for conducting experimentation are also a challenge. Many jurisdictions require the operator to have an appropriate operator license. For example, in Australia, according to the Civil Aviation and Safety Authority (CASA) [115], only trained pilots are allowed to operate the drones at night and must be far from urban areas. This introduces challenges in data collection, trial and testing processes.

Another issue is the resolution limitation of available thermal sensors. The highest resolution radiometric LWIR from FLIR that can satisfy size and weights constraints for small drone applications is the FLIR Tau2 at 640 × 480 pixels. In comparison, RGB Picam v2 [116] has ten times the total pixels at less than one hundredth of the price. Additionally, thermal data contain less information to work with, such as texture, colour or edges [117]. For example, objects with colour textures in colour images will usually appear uniform in thermal data, which results in fewer detected features in thermal imagery.

Thermal images change in appearance over time due to heating and cooling cycles of the scene. Unlike reflected visible light, thermal landmarks data are highly influenced by uncontrolled external sources. For example, as shown in [47], thermal data can be influenced by relative humidity, air convection, reflective surface and other heat sources such as the sun. As a result, thermal landmarks can be vastly different from early morning to late afternoon from which it is reasonable to expect problems with feature based tracking algorithms such as SLAM and optical flow.

Correction conversion techniques such as AGC and FFC/NUC also have a negative impact on image repeatability in re-scaled 8 bit data. Even though there are some proposed workaround methods to overcome this issue, full radiometric data are enormously favoured for producing consistent results. The downside of using full thermal data is it may not work with standard computer vision libraries such as OpenCV. As a result, the development phase can take longer and require more effort. Nevertheless, we strongly recommend future studies to use full radiometric raw data.

## 13. Conclusions

Despite the potential of thermal imaging, limited efforts are being applied to visual navigation to date. Many challenges remain to be fully overcome, including the higher price tag of the sensor, difficulty in obtaining sensors in some parts of the world, thermal landmarks changing through diurnal and seasonal cycles, lack of texture and low resolution, uncontrollable internal image re-scaling and correction.

Thermal sensors can provide valuable information for navigation fusion systems. Thermal sensors can reveal hidden textures behind fog, smoke or darkness and thermal masses underground, which enhance the overall performance of the fusion system in visually degraded conditions.

With the introduction of more portable radiometric sensors, standalone thermal sensor systems can produce good results in visual odometry, VSLAM, optical flow and deep learning. Furthermore, after presenting problems with associated re-scaled thermal data, we highlighted that using full radiometric data should be selected to bypass the re-scaling processes in order to increase the reliability of the system.

The field is not yet mature. Thermal sensors have not been applied in every way that optical sensors have been used. Their different mode of operation of re-radiation rather than reflection has not been exploited apart from offering the ability to see in different conditions than optical sensors. Thermal data changes during the heating and cooling cycles during the day, and this effect remains to be used between seasons. While the environments in which optical sensors are compromised are well known to most humans before even running an experiment, thermal sensors require research and calculation in order to establish where success and failure might occur. As a consequence, thermal sensor strengths and weaknesses for navigation have not been comprehensively considered.

To date, there has been limited effort on researching deep learning techniques that show potential at low resolutions. Considering how successful deep learning and convolutional neural networks in the computer vision field have been, more work needs to be performed in order to overcome the challenges of low resolution in thermal imagery.

Similarly, attention should be given to map-less systems due to their low computational demand and robustness. There is also limited research on fixed-wing aircraft and unmanned ground vehicles. The use of cooled thermal sensors should also be considered due to their superior noise performance and resolution, particularly in cold conditions and low thermal contrast situations.

Our future work will focus on low resolution and low cost thermal sensors as an adjunct to existing navigation systems. We believe this area has the largest opportunity to provide enough value in achieving ubiquitous thermal sensor integration in UAV navigation systems.

## Figures and Tables

**Table 1:** Specifications of thermal sensors presented in this study.

Sensor	Dimension	Weight	Resolution	Fps	Radiometric	Power	Platform	Cost	Released
Thermal-Eye 2000B	282 × 279 × 290 mm	4.54 kg	320 × 240	12.5	No	28 W	UGV	discontinued	n/a
Gobi-640-GigE	49 × 49 × 79 mm	263 g	640 × 480	50	No	4.5 W	UGV	discontinued	2008
Miricle 307 K	45 × 52 × 48 mm	95 g	640 × 480	15	No	3.3 W	UAV	discontinued	2006
FLIR Tau2	44.5 × 44.5 × 30 mm	<70 g	640 × 480	60	Yes	1 W	UAV	$6500 [27]	2015
FLIR A65	120 × 125 × 280 mm	200 g	640 × 512	30	Yes	3.5 W	UAV	$7895 [28]	2016
FLIR Boson	21 × 21 × 11 mm	7.5 g	640 × 512	60	Yes	0.5 W	UAV	$3520 [29]	2020
FLIR Lepton 3.5	10.5 × 12.7 × 7.14 mm	0.9 g	160 × 120	8.7	Yes	0.15 W	UAV	$199 [30]	2018

**Table 2:** Emissivity values for some materials [45].

	Material	Emissivity Value
**Metal**	Aluminium: oxidised	0.4
	Aluminium: polished	0.05
	Brass: oxidised	0.6
	Brass: polished	0.02
	Copper: oxidised	0.71
	Copper: polished	0.03
**Non-metal**	Clay	0.95
	Ice	0.98
	Rubber	0.95
	Water	0.93
	Glass	0.98

**Table 3:** Settings parameters for LK technique and Shi–Tomasi corner detection algorithm [63].

Feature Detection Setting	Maximum corners	1000
	Quality level	0.02
	Minimum distance	5
	Block size	5
LK Settings	Window size	(15,15)
	Maximum pyramid level	2
	Search termination count	10
	Search termination ϵ	0.03

**Table 4:** Summary of presented works.

Work	Sensors Configuration	8-Bit/14-Bit	Sensor Name	Resolution	FPS	Navigation System	Navigation Task
Maddern and Vidas [76]	Combine	8	Thermoteknix Miricle 307K	640 × 480	15	Map-building	Mapping
Brunner et al. [81]	Combine	8	Raytheon Thermal-Eye 2000B	480 × 576	12.5	Map-building	Visual-SLAM
Shin et al. [85]	Thermal only	14	FLIR A65	640 × 512	30	Map-building	Visual-SLAM
Chen et al. [82]	Combine	n/a	n/a	n/a	n/a	Map-building	Visual-SLAM
Mouats et al. [83]	Combine	8	Gobi-640-GigE from Xenics	640 × 480	50	Map-building	Stereo odometry
Mouats et al. [61]	Thermal only	8	FLIR Tau2	640 × 480	30	Map-building	Stereo odometry
Poujol et al. [77]	Combine	8	Gobi-640-GigE from Xenics	640 × 480	50	Map-building	Odometry
Khattak et al. [84]	Combine	8	FLIR Tau2	640 × 480	30	Map-building	Odometry
Khattak et al. [48]	Thermal only	8	FLIR Tau2	640 × 480	30	Map-building	Odometry
Khattak et al. [86]	Thermal only	14	FLIR Tau2	640 × 480	30	Map-building	Odometry
Rosser et al. [63]	Thermal only	8	FLIR Lepton 3.5	160 × 120	8.7	Mapless	Odometry
Choi et al. [104]	Thermal only	8	n/a	n/a	n/a	Deep learning	Thermal image enhancement
Saputra et al. [110]	Thermal only	8	Flir Boson/FLIR E95	640 × 512/464 × 348	60/60	Deep learning	Odometry

## Data Availability

Not applicable.

## Abbreviations

The following abbreviations are used in this manuscript:

LWIR	Long Wavelength Infrared;
AGC	Automatic Gain Control;
NUC	Non-Uniformity Correction;
UAV	Unmanned Aerial Vehicle;
UGV	Unmanned Ground Vehicle;
SLAM	Simultaneous Localisation and Mapping;
TF	Thermal Flow;
OF	Optical Flow;
LK	Lucas Kanade.
